# Homeopathy – what are the active ingredients? An exploratory study using the UK Medical Research Council's framework for the evaluation of complex interventions

**DOI:** 10.1186/1472-6882-6-37

**Published:** 2006-11-13

**Authors:** Trevor DB Thompson, Marjorie Weiss

**Affiliations:** 1 Academic Unit of Primary Care, Cotham House, Cotham Hill, Bristol BS6 6JL, UK; 2 Department of Pharmacy and Pharmacology, University of Bath, UK

## Abstract

**Background:**

Research in homeopathy has traditionally addressed itself to defining the effectiveness of homeopathic potencies in comparison to placebo medication. There is now increasing awareness that the homeopathic consultation is in itself a therapeutic intervention working independently or synergistically with the prescribed remedy. Our objective was to identify and evalute potential "active ingredients" of the homeopathic approach as a whole, in a prospective formal case series, which draws on actual consultation data, and is based on the MRC framework for the evaluation of complex interventions.

**Methods:**

Following on from a theoretical review of how homeopathic care might mediate its effects, 18 patients were prospectively recruited to a case series based at Bristol Homeopathic Hospital. Patients, who lived with one of three index conditions, were interviewed before and after a five visit "package of care". All consultations were recorded and transcribed verbatim. Additional data, including generic and condition-specific questionnaires, artwork and "significant other" reports were collected. Textual data was subject to thematic analysis and triangulated with other sources.

**Results:**

We judged that around one third of patients had experienced a major improvement in their health over the study period, a third had some improvement and a third had no improvement. Putative active ingredients included the patients' "openness to the mind-body connection", consultational empathy, in-depth enquiry into bodily complaints, disclosure, the remedy matching process and, potentially, the homeopathic remedies themselves.

**Conclusion:**

This study has has identified, using primary consultation and other data, a range of factors that might account for the effectiveness of homeopathic care. Some of these, such as empathy, are non-specific. Others, such as the remedy matching process, are specific to homeopathy. These findings counsel against the use of placebo-controlled RCT designs in which both arms would potentially be receiving specific active ingredients. Future research in homeopathy should focus on pragmatic trials and seek to confirm or refute the therapeutic role of constructs such as patient "openness", disclosure and homeopathicity.

## Background

Homeopathic medicine continues to attract attention in medical journals and the media as a popular form of complementary medicine (CM) whose proposed mechanism of action seems incompatible with mainstream scientific thought and the research evidence for which remains controversial. At the centre of clinical research in into homeopathic medication lies the placebo controlled randomised clinical trial (RCT). RCT findings for homeopathic medications are heterogeneous and meta-analytical approaches have reached both positive [1] and negative conclusions [2].

An assumption underlying RCT-based research is that homeopathic medicines can be considered as a special class of pharmaceutical agent with specific effects. When, as in the Shang et al meta-analysis,[2] the therapy is presented as having no specific effects, it is considered to be mediated by "non-specific" effects. An intervention based solely on such effects is considered of little value – the editor of the Lancet proposing that Shang's findings heralded the "end of homeopathy". But despite these condemnations, homeopathic care remains popular, a fact which some accord to the empathic style of homeopathic practitioners.

The RCT model is only one possible way of approaching the riddle of homeopathy – other include in vitro studies [3] and large-scale observational studies [4-7]. In this study we consider it not as a pharmaceutical intervention, but as a *complex *intervention and have analysed its active ingredients using a framework proposed by the UK Medical Research Council (MRC) [8]. This treatment is justified because the homeopathic approach contains "a number of components which may act both independently and interdependently" – the criterion for the definition of a complex intervention. For instance consultations involve the patient in an unusally detailed exposition of their complaints, an attentive practitioner and a process of matching between the patient's predicament and what is known of a wide range of homeopathic medicines. Thus even on prima facie grounds there are a number of potential factors at play.

The first stage of the MRC framework asks the investigator to explore how a given intervention might, *in theory*, be beneficial. Here we worked on the assumption that it is unlikely that the effects of homeopathic care are conveyed by unique mechanisms. In other words, broader psychological and anthropological theories of healing should contribute to our understanding of homeopathy. We explored literature on the placebo effect[9], universal anthropological models [10], psychotherapeutic practices [11] and psychological models such as disclosure theory [12]. We also took expert opinion from practitioners in fields such as gestalt and narrative therapy [13]. We used this knowledge to sensitise us to processes revealed in stage two of the MRC framework, which involves the direct observation and modelling in a real-world context. The purpose of this modelling is to "identify the components of the intervention and underlying mechanims by which they will influence outcome".

In this paper we present the findings of formal case series approach to the study of 18 patients referred for care at Bristol Homeopathic Hospital (BHH). This is the first time that the *process *of routine homeopathic care has been the focus of systematic qualitative study. By defining potential active ingredients in this way we hope to shed important light on the concept of "non-specific" effects and provide a more nuanced approach to the workings of homeopathy than can be found in the RCT.

In summary, our objective was to identify and evalute potential "active ingredients" of the homeopathic approach in a prospective formal case series, which draws on actual consultation data, and is based on the MRC framework for the evaluation of complex interventions.

## Methods

We have described elsewhere the case series methodology used here [14]. Case studies should be used to "investigate a contemporary phenomenon within its real-life context especially when the boundary between phenomenon (*the remedy*) and the context (*the consultation*) are not clearly evident" as we consider to be the case with homeopathy [15]. Case studies rely on the use of "multiple sources of evidence with data needing to converge in a triangulating fashion" and appeal to reason rather than statistical inference [15].

From January to May 2003, all consecutive referrals to Bristol Homeopathic Hospital (BHH), living with one of three index conditions, were contacted and invited to join the study. The three index conditions were irritable bowel syndrome (IBS), chronic fatigue syndrome (CFS) and childhood atopic dermatitis (AD). Of the 33 such patients, six did not respond to the initial and one follow-up mailing, two replied but declined to join the study, and six agreed to join but did not fulfil the study criteria. Inclusion and exclusion criteria included that the index condition should be their main complaint, that they were not already receiving homeopathic treatment and that they were not engaged in any other research study of the index condition. In addition, for AD they needed to score at least five on the Children's Dermatology Life Quality Index (CDLQI) [16], as children with trivial eczema are sometimes referred in whom it is difficult to show a treatment effect. Recruitmented ended with the consent of 18 full participants. Details of these 18 patients are included in Table 1. All names are pseudonyms.

**Table 1:** Description of the 18 participants of the case series at Bristol Homeopathic Hospital.

**Unique ID**	**Study Name**	**Diagnosis**	**Age/Gender**	**Global Outcome (GOA)**	**Referral Initiation**	**Previous Hom?**
001	Emily	IBS	37 F	Major	Patient	No
003	Jack	CFS	46 M	Other	Consultant	No
008	Joshua	IBS	65 M	None	Patient	No
009	Ellie	CFS	42 F	Some	Patient	Yes
010	Thomas	AD	6 M	Some	Parent	No
012	Jessica	AD	15 F	Major	Parent	Yes (Mum)
013	Sophie	CFS	56 F	Major	GP	Yes
015	Chloe	IBS	21 F	None	Nurse	No
016	Lucy	IBS	59 F	Major	Patient	No
017	Olivia	IBS	48 F	Major	Patient	No
018	Charlotte	CFS	27 F	Some	Patient	No
020	James	AD	4 M	None	Parent	No
023	Katie	CFS	47 F	Some	GP	No
025	Grace	AD	8 F	Some	Parent	Yes (Mum)
027	Hannah	AD	10 F	Some	Patient	No
030	Leila	CFS	60 F	Major	GP	No
032	Lily	AD	5 F	Some	Parent	No
033	Emma	IBS	46 F	None	GP	No

Each patient agreed to attend a "package of care", consisting of an initial consultation and four follow-up appointments over an eight month period, with one of three allocated homeopathic physicians. This is the standard package that BHH offers, though at BHH there is typically a longer (3–6 month) gap between later consultations. Before the start, and at the end of the package of care, patients were interviewed by the principal investigator (TT). At recruitment, initial consultation and around the time of subsequent consultations, participants filled in condition-specific and generic outcome measures. At the end they nominated a "significant other" to report on their progress and were asked to produce a piece of artwork reflecting on their care. The volume and diversity of data types allowed for the triangulation of multiple perspectives but also limits the size of sample that can be practically managed [17].

Interviews were semi-structured around a topic guide. The initial "entry" interview focused on patient expectations and the "exit" interview on understanding patients' experience under care. The main interview topics for each interview are included in Table 2. All interviews and consultations were recorded and transcribed verbatim. Condition-specific measures comprised the Irritable Bowel Syndrome Quality of Life Scale (IBSQoL) [18], the Fatigue Impact Scale for CFS (FIS) [19] and CDLQI for AD. Generic measures comprised the Measure Yourself Medical Outcome Profile (MYMOP) [20] the Consultation and Relational Empathy Scale (CARE)[21] and the Glasgow Homeopathic Hospital Outcome Scale (GHHOS) [22]. Scoring of outcome by physicians was also recorded. Such scoring, on a scale from +3 to -3, is a routine part of activity analysis at BHH. The results on the scoring of 6544 consultations have recently been published [7].

**Table 2:** Principle topics for "entry" and "exit" interviews

**"Entry" Interview Topics**
Prior experience of conventional treatment for index condition
Referral pathway to BHH (e.g. initiated by whom?)
Prior experience of homeopathy (self, family, friends, media)
Attractions of homeopathy
Expectations about content of first homeopathic consultation
Thoughts on the "mind body link" in relation to their problem
Expectations about outcome of homeopathic treatment
Thoughts on how homeopathy "works"
**"Exit" Interview Topics**
Process of care
Experience of homeopathic treatment
Insights into self and health through care process
Comparison with other health care experiences
Comparison of prior expectations with perceived reality of care
Experience of personal attributes of homeopathic doctor
Remedy
Beliefs on how this was chosen
Understanding of the nature of the remedy
Experience of taking the remedy
Outcome
Health changes over treatment period
Health changes in relation to other measures such as questionnaire scores
Views on relative influences of consultations, remedies, chance etc

Given the small sample size, the quantitative questionnaires were not analysed statistically but were compared with qualitative findings. The qualitative data was coded in Atlas.ti QDA software and analysed by emergent and pre-existing themes derived from prior theoretical study in which theory and data analysis are produced dialectically rather than inductively as in Grounded Theory [23]. Analysis was guided by Jennifer Mason's text "Qualitative Researching" [24]. A subset of consultation and interview transcripts were blindly coded in parallel by MW and coding themes compared. Special attention was paid to discomfirming cases – i.e. those cases which tended to contradict any emerging consensus.

The study received ethical approval from the Research Ethics Committee of the United Bristol Healthcare Trust (UBHT), Bristol, UK in December 2002 (study reference E5502). Data collection continued until May 2004. Informed, written consent was obtained from patients and the carers of child participants. In the majority of cases signed consent was additionally obtained from children.

## Results

### Estimating Outcome

This is a study of the *active *ingredients of the homeopathic process. To understand such activity, it is helpful to relate it to the outcome (positive or negative) of the therapy. This study was not designed to prove, by statistical inference, the outcome of the homeopathic "package of care" or the contribution of the various ingredients. However, it is possible, using the sorts of inference that clinicians use routinely, to make some judgement about the changes in health status of the study participants over the treatment period. In keeping with the case series methodology, this is done by triangulation of data from a range of different sources as listed in Table 3. Patient consultation and interview narratives were compared to their artwork, significant others reports, numeric outcome data and physician scoring to create an informal composite measure termed "global outcome assessment" or GOA. GOA places patients into three categories of "Major", "Some" and "None" in terms of changes in health status over the treatment period.

**Table 3:** The data sources used to judge outcome in the case series

1. Qualitative Data from Homeopathic Consultations
2. Qualitative Data from Exit Interviews
3. Patient Artwork
4. Significant Others Reports
5. Numeric outcome data including GHHOS, MYMOP, IBS-QoL, FIS and CDLQI
6. Physicians outcome scoring

**Figure 1:**
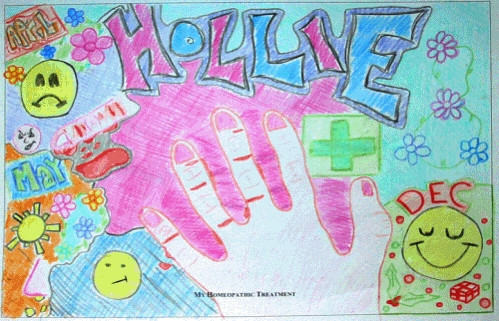
Artwork from Jessica in Global Outcome Assessment category "Major".

**Figure 2:**
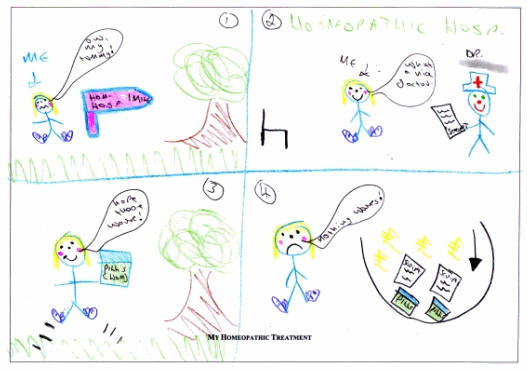
Artwork from Chloe in Global Outcome Assessment category "None".

The volume of data required to show how all GOA assignations were made is beyond the scope of this paper. The procedure can be exemplified with findings from two participants from GOA category "Major" (Jessica) and "None" (Chloe) respectively. Compare first of all the artwork of Jessica in Figure 1 with that of Chloe in Figure 2.

Both show the different phases of the therapeutic process – but whilst Jessica's finishes in December with a happy face, Chloe's ends in what she calls at interview a "black hole" with the epithet "nothing works". Next compare the significant other's report written by Jessica's mother:

[012:Jessica – AD]. Significant Other Report [mother]

"When Jessica started her treatment, her right hand, especially the knuckles were very sore and the skin cracked and bleeding. She found it difficult to do many simple things, even washing was a problem, as water seemed to make the symptoms worse. She was unhappy and her appetite was poor. Since she started Homeopathic treatment her condition has improved significantly – even clearing up completely for several weeks. This of course stopped the 'itch/scratch' situation. Jesscia's appetite has greatly improved, she can bathe or shower without pain. She has also started swimming again, something that she found very painful. For the first time in years Jessica's right hand is free from eczema and she is a lot happier."

with that written by the boyfriend of Chloe:

[015:Chloe – IBS] Significant Other Report [boyfriend]

"Over the last eight months of Chloe's treatment I have noticed no significant change to the condition of her IBS. Apart from regular exercise and a strict lifestyle routine nothing seems to affect her problem. Some days she is worse than others and I can see how frustrating this problem is, it is very disheartening. It is often more uncomfortable towards the end of the day and can be quite restrictive. It as much effects her physically as it does emotionally. It seems like she has almost come to accept the problem as nothing prescribed medically touches the condition. It still is a problem for her and there should be a solution! As regards to her homeopathic experience however, Chloe has always reported a friendly service."

**Figure 3:**
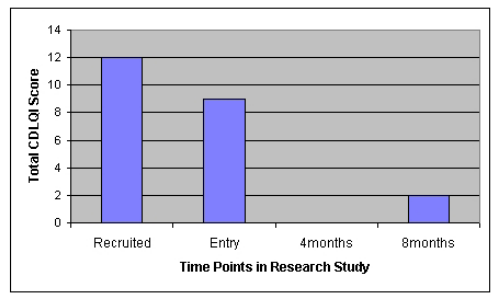
CDLQI of Jessica in Global Outcome Assessment category "Major".

**Figure 4:**
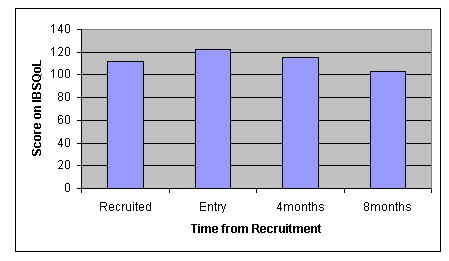
IBSQoL for Chloe in Global Outcome Assessment category "None".

Finally look at the CDQLI for Jessica in Figure 3 and the IBSQoL results for Chloe in Figure 4, where higher scoring signals a worsening of the condition.

**Figure 5:**
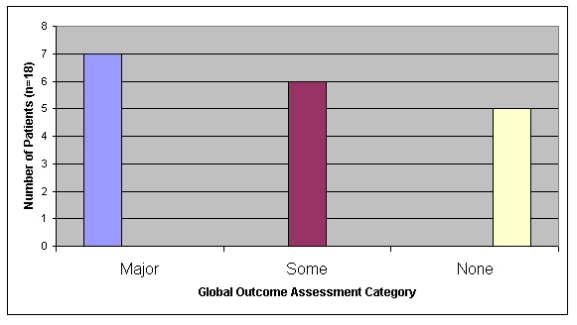
Global Outcome Assessment for the 18 cases.

By triangulating data for each case, assignations for the 18 cases were made as shown in Figure 5. According to this reckoning, seven cases experienced a major change in health status, six some change and five no change, over the eight month treatment period.

The authors make no claim to have conclusively demonstrated that the homeopathic intervention is responsible for these health changes. In one case of GOA "major", (003 Jack CFS), the patient was manifestly improving *before *the start of the treatment period and this improvement persisted throughout. However in the other six GOA "major" cases it is our opinion that the homeopathic approach was involved in facilitating the health gains experienced by the patients. *How *that aid might be mediated is an open question that is explored in the remainder of this paper.

### Introducing the Active Ingredients

Common sense would suggest that there are many ways in which the effects of the homeopathic approach could be mediated. In our research we do not propose to offer a *systematic *approach and to include all the different modes of action that have been postulated. For instance we do not consider complexity [25] or quantum models [26]. Indeed the whole body of prior research on the therapeutics of interpersonal communication would be of potential relevance. Instead we present data on those active ingredients that emerged as most interesting in the course of our theoretical and empirical work, hoping to open up a field for further investigation.

In the following sections we look at the influence of psychological type (and in particular the construct of "openness"), consultational empathy, disclosure phenomena, narratological aspects, homeopathicity and the specific role of the remedy. We begin however where the patient begins, with an examination of their expectation that homeopathy will be of benefit. In what follows, quotations are identified with participant number, pseudonym, diagnostic group, GOA category and data source. Data sources include entry and exit interviews and any of the patient's five homeopathic consultations.

### The role of patient expectation

Classical placebo theory predicts that patients' responses to an intervention are based on their expectation of likely benefit – their "belief" in the treatment. For instance branded aspirin is more effective than pharmaceutically identicle generic aspirin [27]. By extention is has been argued that patients benefit from homeopathy because they believe in it. At recruitment, all patients were optimistic. It would be curious if they were not optimistic. Their optimism was often based on what they had heard of the success of other patients (12/18) or on personal experience (4/18). In most cases (13/18), optimism was not based on any clear understanding of the system – this quote is typical:

I don't really know a lot about homeopathy. I only know that there's only a small amount of a particular thing in the solution, it gets reduced down, that's about all I really know about it. [018:Charlotte. GOA:some. *Exit i/v*]

Most then were relatively uninformed. Every patient was asked if they thought homeopathy would help them but we could detect no obvious relationship between level of pre-treatment expectation and final GOA outcome.

Expectation of benefit is also generated *during *consultations. We specifically identified the ways in which practitioners framed the likelihood of benefit. In this sample this was done in a way that was congruent with the presumptive aims of a therapeutic process – this quotation is typical:

Okay. ... Well ... I think homeopathy should be able to help you. ... I think we should be able to get this pain ... at a better level ... at a more manageable level so that ... so that you're more able to be active. [*in *008: Joshua – IBS. GOA:none. *initial consultation*]

However derived, most patients came away from their initial consultations with a sense of hope that their chronic ailments might be relieved. For most this contrasted with their experience of conventional care:

Whereas when you go to ... a GP, they sort of, they give you the creams and things but it's almost like 'oh that's something you've got to live with.' They don't really give the impression that there will be a cure. [010:Thomas – AD. GOA:some. *entry i/v*]

We know that hopelessness is a nocebic state of mind [28] and it seems likely that homeopathy's ability to engender hope is one of the ways its effects could be mediated.

### Openness to the mind-body connection

The homeopathic worldview favours an integration of mind and body and one might predict that patients who come to homeopathy open to this dynamic will fare better than those expecting a conventional pharmaceutical approach. Literature exists confirming an association between the psychological construct of "openness" and the tendency to engage with CM therapies [29]. These findings and others, [30] open up the interesting idea that not only are there alternative therapies but also alternative *patients *who bring with them a predisposition to respond to CM.

At entry interview, nine patients contemplated a mind-body link in relation to their own health problem. These nine included all but one of the patients with major health gain. These findings may conform with those of Bell et al, [31] which shows better outcome in individuals in a trial of homeopathy for fibromyalgia displaying the trait of "absorption" which is highly correlated with the construct "openness" described above.

This propensity is therefore a strong candidate for an active ingredient – though there are several qualifications. Jessica, for instance, described herself being initially perplexed by being asked about her emotional life:

I couldn't really understand, I thought it would be all about making things better, be like assessing the suffering to my fingers, I didn't think any of it could be to do with emotional things. [012: Jessica – AD.GOA.major. *exit i/v*]

In retrospect Jessica felt the addressing of the emotional side to her case was an essential part in her care (see below). Patients may come along unaware of the mind-body premises of homeopathy, but learn fast through experience. Conversely Chloe [015] was open to the psychological dimension:

What do I expect? ... I expect him not just to look at the bowel problem, as such, but also the medication I've taken long-term in the past and also those family situations which I'm sure had a bearing ...in the first half of the nineteen nineties. [015: Chloe – IBS.GOA.none. *entry i/v*]

But this hint was not actively pursued in the consultation and is a potential explanation for a striking lack of therapeutic success in her case. Being open to the mind-body connection may be necessary but it is not sufficient – those links must then be pursued by the practitioner. It may not be that this openness predisposes patients to respond but rather that such openness gives the physician the insights necessary for accurate remedy selection.

### Consultational Empathy

The empathic nature of homeopathic consultations is often cited as the reason why patients appear to respond:

the long interview – about an hour-and-a-half – carried out by an empathetic practitioner during diagnosis may explain why people report improvements in their health. [32]

Mercer has established that patients experience empathy within homeopathy consultations [33]. In defining a quantitative measure, Mercer has described empathy according to a range of criteria [34]. Here this five year old describes, for instance, the feeling of "being listened to":

TT: How did you find the doctor, Lily, to talk to?

032:... It was nice to talk to ... what s/he does is, when anyone says anything s/he listens and when s/he's in the middle of talking ... s/he just stops and listens.

TT: Right. ... And does that feel good? ... When your doctor does that?

032: Yeah it feels really helpful 'cos whenever I want to tell him/her something, s/he just stops and listens [032:Lily – AD. GOA:some. *Exit i/v*]

As well as getting the chance to speak and be listened to, according to Mercer, patients also wish to know that their individual experience has been properly understood and recorded by a caring doctor – a phenomenon well articulated by this CFS patient:

S/he never put any words in my mouth or tried to change what I said. S/he would seem to just say a sentence or two which encapsulated all what I was trying to tell him/her, which was just really amazing, I mean it showed how much s/he was listening to me and how much s/he wanted to help me get well. [013:Sophie – CFS. GOA:major.*Exit i/v*]

There was one case where there seemed to be a failure of empathy or certainly of rapport – in which, after a very promising start, the patient and parent seemed to disengage with the process. At exit interview, this disengagement was found to be due to not feeling at ease with the doctor:

027b (mother): I'd say ... if Hannah could put it into words, s/he [*means the doctor*] seemed a bit *detached*, I would say. For like an hour long interview with a child ... I would say s/he was quite detached.

027: I would have preferred if it was a different doctor. [027:Hannah – AD. GOA:some. *Exit i/v*]

As Mercer has shown in other contexts, there was no good outcome without satisfactory empathy, but good empathy was not itself sufficient for good outcome [35]. Paradoxically outcome here did not match scoring on the CARE scale. Doctor empathy at BHH was rated at around the average for GPs in Scotland, but six out of the seven GOA "major" patients rated their doctors' empathy at *below *average for the BHH sample. This may be a chance finding due to the small sample size but does not provide evidence that higher empathy links to better outcome. Such a linkage was not determined in recent findings from the Glasgow Homeopathic Hospital [36].

### Narratology of Homeopathic Care

All medical consultations are "co-constructed" by patient and practitioner. For instance their content depends on what the patient feels comfortable to reveal and on the type of story the practitioner feels it is relevant to elicit. The consultations in this study involved the patients in voicing multiple aspects of their inner experience which we term here "Lifeworld" after Mishler [37]. This contrasts with the findings of Barry et al, in which, in 11/35 UK primary care consultations, the voice of the Lifeworld was absent [38]. Patients described how in the conventional setting the wider picture (Lifeworld) can be suppressed:

It [conventional medicine] has its place, however it doesn't look at the whole person, it just focuses on the physical body and doesn't look at the emotions, the psychology, the make-up, the lifestyle of the person and so doesn't see the whole picture and it's like, I feel orthodox medicine compartmentalises and just focuses on what's the actual problem without necessarily seeing where that problem has come from. [013:Sophie – CFS. GOA:major. *Entry i/v*]

"Time" with the practitioner is often cited as an active ingredient in homeopathy. As with empathy, it is unlikely that without sufficent time, homeopathy would work. For instance the homeopath needs certain information in order to choose the remedy and that information takes time to emerge. But this data goes against the idea of it being "just time" because of the particular way in which homeopathic consultation time is used.

Of conventional medical interviews, the psychiatric clinical examination has similarities in structure to a homeopathic interview. Both last around one hour, cover the presenting complaint and include exploration of biography, social history and past medical history. In addition to these features, homeopathic consultations include elements that are relatively unique and specific to homeopathy.

For instance, particular importance is attached to the identification of peculiar incidental bodily and psychological symptoms. For example Leila's (030 – CFS. GOA:major) fear of *birds *guided her doctor's choice of medication. Peculiar systems, which would lack relevance for conventional diagnosis, are very useful in homeopathic (remedy) diagnosis.

Another is the way in which practitioners ask for a level of detail about bodily symptoms that would never been seen in conventional practice:

Doctor: So just go into that feeling, that sensation, that experience. ... Something "really tight crushing something to break it". Give me other images that come into your mind when you think of that. [*in *018:Charlotte. GOA:some. *4*^*th *^*FU cons *^*n*^)

This combination of intense interest in the symptoms of the body coupled with imaginational prompts is resonant with "focusing" – a branch of psychotherapy [11]. Similar probing occurred with psychological symptoms:

Doctor: And tell me again what that feeling was when you looked at the board and you didn't know what had happened, what was that feeling? [09:Ellie – CFS. GOA:Some. *Initial cons *^*n*^]

This probing, and the sort of information that came out as a result of it, has led us to divide Mishler's concept of "Lifeworld" into "superficial" and "deep" aspects. Deep aspects might include revelations of past traumas, existential crises, relationship conflicts and any material that might be held as poignant and highly meaningful in the context of the person's life. We postulated that it was the revelation of such material (more likely in those showing traits of "openness", see above) would constitute a specific active ingredient of homeopathic care.

In three of the seven "GOA:major" cases, expressions of deep aspects of the Lifeworld were encountered in need, one might say, of "healing" (see section on disclosure below for an example). However in two of the "GOA:major" cases, no deep Lifeworld was revealed. Expression of deep Lifeworld would not appear to be necessary for therapeutic benefit.

There was one example in which the homeopathic approach conspicuously failed to benefit a patient living with IBS (015:Chloe – IBS. GOA:none). Here intimate aspects of Lifeworld were alluded to by the patient but not overtly followed up by the doctor. In another case of treatment failure, the *patient *appeared to *block *expression of deep Lifeworld:

Doctor: Now I must admit to be feeling quite challenged in this case. I always feel tense, I always feel when I try and go in that I'm pushed out, so that, for instance, if I go to something that's slightly emotional she'll often say 'It is no longer an issue' which kind of blocks things, makes one uneasy to go deeper. [033:Emma – IBS. GOA:none.*3*^*nd *^*follow-up cons *^*n*^]

It might be that where unexpressed deep Lifeworld exists it has to be expressed for homeopathic healing to function. Where the medical gaze does not encompass that deep Lifeworld, or the patient is unwilling to share it, it becomes harder to get a response. But again an interpretive problem emerges. Is it the revelation of deep   Lifeworld that is curative in itself or is it the fact that the information   revealed from the deep Lifeworld allows the homeopath an insight that leads   to a more accurate prescription? In the following sections we explore perspectives that would suggest either of these to be possibilities.

### Disclosure

Disclosure theory sets out that a person's sense of identity is fractured by traumatic events which "threaten the temporal and structural coherence of one's self-story" [39]. Disclosure helps the person to gain insight into his or her trauma and in so doing can repair damaged self perceptions and engender a more resilient self-concept [40]. In several cases in this series, traumatic material was disclosed. The most vivid example is that of Jessica [012]. Jessica's father had suffered a brain injury before her birth and was mentally and physically handicapped through to his early death when Jessica was five or six. He liked to take her to school but, perhaps due to frontal lobe damage, was unaware of his habit of holding her hand so hard that it hurt. Jessica asked her Dad if her Mum could take her to school instead of him. Soon after this time he died. Without ever telling her mother or anyone else, Jessica had lived the ensuing decade with the guilt of having rejected his attention. In the homeopathic consultation she disclosed her feelings for the first time:

I think me talking about everything that was like, I had bottled up and things that were affecting my life, I just think that really helped take a big weight off my shoulders and just to find someone I could talk to and that could understand and then tell me that it weren't my fault, without feeling stupid or silly, like I've done something wrong. [012: Jessica – AD. GOA:major. *Exit i/v*]

In this case it is tempting to speculate that Jessica's disclosure was an active ingredient in the mediation of the marked health gains she experienced.

### Homeopathicity

**Figure 6:**
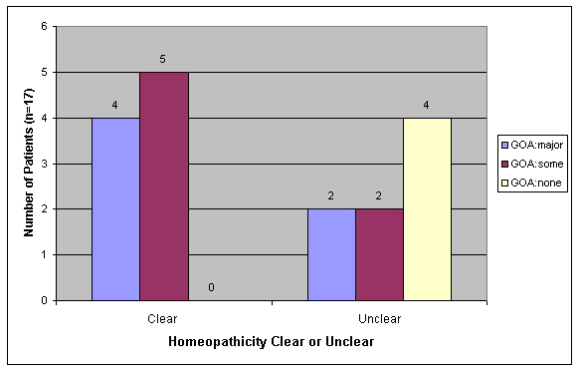
Chart showing *homeopathicity *by Global Outcome Assessment category.

A specific part of the process of homeopathic care is the way in which Lifeworld is matched with what is known about homeopathic remedies. This matching is, in the eyes of the homeopathic community, the most important of all active ingredients. Improving ones matching ability is the main thrust of postgraduate homeopathic education. A variety of frameworks for this matching were used by the doctors which are beyond the scope of this paper to describe. There is an absense of evidence that different practitioners will concord in their prescribing choices in any given case [41]. Demonstrating a link between homeopathicity (the accuracy of the match between Lifeworld and homeopathic materia medica) and outcome would be an important topic for future research. In this study such a link can be demonstrated only retrospectively. We divided prescriptions into two groups "Match:clear" and "Match:unclear". "Match:clear" meant we could determine a clear link between what was revealed in the consultations and the choice of homeopathic remedy. In "Match:unclear" the connection was hard to determine. These groups were plotted separately against the three GOA outcome categories. See Figure 6. These results suggest a possible link between homeopathicity and outcome, worthy of further investigation.

### The Remedy as Active Ingredient

The relative contributions of remedy and context cannot be distinguished either in real-world homeopathy or in this study. Most homeopaths behave *as if *the remedy is the main active ingredient. In this study, in addition to the elements outlined already, we identified a range of phenomena which were compatible with the specific action of the homeopathic remedy.

There were, for instance, in this series, no examples of the patients' health status improving *before *taking the presecribed remedy (though this phenomenon has been recorded anecdotally). Patients often described clear dose dependent beneficial effects:

I do feel different and quite quickly afterwards. I can honestly say that within a couple of hours I do feel different, calmer, more relaxed, more in control. [001:Emily – IBS. GOA:major. *3*^*rd *^*FU cons *^*n*^]

Perhaps more suggestive is amplification of presenting symptoms soon after taking the medication – a phenomenon termed the "homeopathic aggravation" and experienced by 9/18 patients in the study – a higher figure than that previously reported [42].

First time I took the remedies my eczema wasn't too bad was it? [*poses question to mother*] it was quite fair, it was still red and angry but first time I took it, it really flared up and just my knuckles swelled up and it was really aggravating, and after about four days it gradually got to get better. [012: Jessica – AD. GOA:major. *exit i/v*]

There is no commonly recognised equivalent to this phenomenon in conventional medicine – though paradoxical effects have been recorded [43]. Such aggravations would be predicted theoretically according to the principle of "like cures like". Another phenomenon, predicted theoretically is the "proving symptom" – in which the patient develops symptoms associated with the prescribed remedy but previously unknown to the patient. There was one striking example of this in the dataset when the 15 year old Jessica, normally a lusty eater, reported a new symptom of feeling full after eating a few mouthfuls after first taking the medicine "Lycopodium". A keynote of this remedy is "easy satiety". This idiocyncratic symptom passed off after a couple of weeks not to return.

Another interesting phenomenon is where the patient responds slightly or not at all to an initial remedy but well to a subsequent choice – this would be more suggestive of specific than a non-specific effect. This happened in 6/18 cases, and is a well reported among homeopaths:

That's when [*means after the prescription of Alumina Silicatum – her third new remedy*] I noticed that there was more change, it felt more significant then, as if the right button had been pressed by the remedy. [013:Sophie – CFS. GOA:major.*Exit i/v*]

These various phenomena give suggestive evidence of the remedy as an active ingredient and complicate the assumption that homeopathic healing is placebo healing – as the homeopathic placebo would appear to have some unique features interwoven with the taking of the homeopathic remedy.

## Discussion

The aim of the current study was to conduct an exploratory investigation into the potential active ingredients of homeopathic therapy using a formal prospective case series approach in accordance with the MRC framework. Its rationale stemmed from the mismatch between, on one hand, the strong clinical effects claimed by homeopaths and recorded in observational studies and, on the other hand, the meagre "true drug effects" in placebo-controlled RCTs.

Homeopathy's detractors do not claim that homeopathy is without clinical effect but that this effect is due to "placebo", "non-specific" or "context" effects, which are generic aspects of the practitioner-patient encounter. Hitherto the nature of such effects has only been systemically investigated in interview studies. We gained unique access to the therapeutic process by recording and analysing the authentic homeopathic *consultations *of 18 patients, each living with one of three distinct index conditions, receiving a five-visit "package of care" at Bristol Homeopathic Hospital. We also collected a range of other types of data including pre and post treatment interviews.

In order to address the question of *active *ingredients, we had to make some estimation of patients' health status at the end of the "package of care". By thorough triangulation we feel confident in our conclusion that roughly a third of patients experienced major health gain, a third some gain and a third no gain over the treatment period. Of the outcome measures we used in our assessment, patient artwork (and patient commentary upon that artwork) was the most revealing and will be reported elsewhere. We cannot conclude that these health gains were *caused *by the homeopathic therapy but in most cases this is a reasonable working hypothesis. The remainder of the study was concerned with looking at factors within the homeopathic process that could have had bearing on these outcomes.

A possible physicianly stereotype of the homeopathic patient is as a person, most likely female, with an empassioned belief in homeopathy tagged onto a range of other "New Age" predelictions [44]. The patients (and parents) in the study did not fit this stereotype. The majority had only a rudimentary understanding of the system and were driven by the pragmatic goal of symptom improvement. Of the 12 adult patients, half had been referred at the suggestion of a health care professional. Patients had expectations of benefit but their levels of expectation did not seem to link with outcome and it was possible to have had high expectation without eventual benefit.

One entry-interview trait that seemed to correspond with good outcome was "openness to the mind-body connection". This was present in all but one of those with good outcome but raises an interesting intrepretative point – did such openness allow patients to respond psychotherapeutically or merely allow the homeopath the diagnostic insights necessary to make an accurate prescription?

On patient-generated ratings of consultational empathy, BHH doctors' average was slightly lower than that of GPs in Scotland. In the one case of apparent breakdown in rapport there was otherwise unexplained clinical failure. These data suggest that empathy is necessary for good outcome but there was no correlation between empathy levels and outcome. Indeed, peculiarly, the opposite was true. Those that did well clinically rated their doctors as less empathic within this small sample. These findings do not enforce the widely held view that it is the empathic nature of the consultations that governs their success. Perhaps in the focused pursuit of remedy diagnosis, and the associated plumbing of "deep lifeworld", patients may feel uncomfortable in the short term. However from the consultation data the impression is of high levels of empathy, which is something to commend the homeopathic approach – even if it not something specific to it.

The concept of "non-specific" implies that an active ingredient is generic and could potentially arise in any clinical encounter. There were aspects of the homeopathic consultations that were by contrast relatively *specific *to homeopathy. For instance, the homeopath's attention to idiopathic symptoms (of no significance to conventional diagnosis) and their intent to elicit detailed and nuanced histories of bodily sensations. These two have no direct equivalent in conventional history-taking though are resonant with fields of psychotherapy.

The most specific aspect of the homeopathic process is of course the homeopathic remedy and these data show that closeness of matching may correspond with outcome. This could be due to the action of the correctly matched remedy or reflect the fact that the practitioner developed a very clear (and therefore therapeutic) understanding of the person's situation – an understanding reflected in "accuracy" of the remedy choice. By either interpretation, it is beyond reasonable doubt that the process of remedy selection has a large impact on the consultation process and constitutes something relatively unique to homeopathy, including homeopathy's ability to prescribe individually from a range of three thousand or more remedies from all kingdoms of nature.

Findings here are consistent with the theories of symbolic healing put forward by Dow [10]. According to Dow traditional healing and western psychotherapy have roughly the same structure. In each the suffering patient attends the healer/therapist who listens to their lament and persuades them it can be understood in terms of a shared cultural myth. The healer/therapist then attaches the patient's emotions to "transactional symbols" particularised from the general myth and manipulates the symbols to allow the patient to "transact" these emotions. In the case of homeopathy the transactional symbol would be the remedy, which is manipulated through the expectations applied to it and the intricacies of the prescribing regimen. The further the homeopath goes into the state of the patient, the more apposite the symbol will be and the more powerful will be the healing response. This model might dispense with the idea of the remedy having any *biological *function, though would still depend on its authentic prescription. But could the remedies also be biologically active?

Half of the 18 patients experienced homeopathic aggravations, one experienced an apparent "proving" symptom and several seemed to respond to later remedies but not to earlier remedies. Against the remedy theory of homeopathy is that some patients responded initially to seemingly well-indicated remedies but failed to respond to subsequent repetition of the same remedy. This would be more typical of a placebo-based mechanism but within the homeopathic framework might be explained as a remedy choice which is only a "partial similar" and therefore not of lasting benefit.

This study has several important limitations. In trying to take a broad exploratory look at the homeopathic phenomenon, it has chosen to study a small number of cases in depth. Findings are not open to statistical testing and it is not practical to quote all the original data in support of each presented finding, for instance with respect to outcome. Because data derived from consultations and semi-structured interview is not systematically generated the quality of data on each topic is variable and may be altogether absent. This effects the way in which causal inferences are drawn and means they can only be suggestive and in need of further qualification and validation in larger studies looking a fewer variables. As this is primarily a study of process (rather than outcome) we believe the absence of a control group is not an inherent weakness.

The principal investigator (TT) is employed as a homeopathic physician by the UK NHS as well as as a family physician. This means that the study is cast from the perspective of someone who believes that homeopathy can be a catalyst for improving health. This role also provides the PI with a particular insight into the homeopathic process that those wed to other methodologies might not naturally embrace. It is our opinion that clinicians can fruitfully research their own field especially in collaboration with others with little or no direct involvement [45]. MW had no prior personal experience as a practitioner, researcher or patient of homeopathy.

The challenge of gaining transparency in these findings is compensated for by the fact that, perhaps for the first time, the actual practice of homeopathic medicine is being subject to naturalistic enquiry. For instance instead of relying on a preselected numerical measures, here the live consultation transcripts, patient artwork and the views of "significant others" were triangulated along with a range of other measures. This, in our opinion, gives an account with much higher external validity than is typically seen in research on homeopathy.

These findings have implications for the future of homeopathic clinical trials. We have demonstratred that the consultational activity within homeopathic care has aspects which are specific to homeopathy. If these aspects are therapeutically active, which is a reasonable working hypothesis, then comparison of placebo and non-placebo arms in homeopathic trials will not constitute a fair test. This is because the patients in the placebo arms will be receiving an active and specific part of the homeopathic care – of which the remedy matching process is a key example. We struggle to understand how the "physical" remedy and the consultational process in which the remedy diagnosis is made, can be separated for the purpose of analysis. Obviously an authentic remedy cannot be prescribed without an authentic consultation, though it has always been argued that you can have the consultation without the remedy. But, like Paterson, we argue that the connection is too complex to be considered with a simple summative model [46]. Better then to compare homeopathy not with placebo but with standard or alternative care using pragmatic designs [47].

For us, some key research to follow on from this study would be into the concept of "homeopathicity". If homeopathy is truly a non-specific intervention, there should be no correlation (perhaps as judged prospectively by a group of homeopathic experts) between the patient's clinical history and the features of the chosen homeopathic remedy (homeopathicity) on one hand and clinical outcome on the other. The demonstration of such a correlation would suggest there was something specific about homeopathy (even if not telling us if this was something consultational or remedy-mediated). Additional promising research topics include other potential active ingredients such as "disclosure" and "openness to the mind body connection".

## Conclusion

This is one of the first studies to open up the black box of the homeopathic process and identify some of the factors that might be responsible for its apparent clinical effects. A range of potentially therapeutic consultational factors was identified, some of which were generic and others, such as the remedy matching process, specific to homeopathic care. Additionally there were phenomena, such as symptom aggravations, that are compatible with a specific action of homeopathic remedies. We conclude therefore that homeopathy can justifiably be considered a complex intervention. This should be taken into account in the design of clinical trials of the therapy and open up a field of research to further characterise these specific and non-specific effects.

## List of Abbreviations

AD Atopic Dermatitis

BHH Bristol Homeopathic Hospital

CM complementary medicine

CARE consultation and relational empathy (scale)

CDLQI children's dermatology life quality index

CFS chronic fatigue syndrome

FIS Fatigue impact scale

GOA Global outcome assessment

GHHOS Glasgow homeopathic hospital outcome scale

IBS Irritable bowel syndrome

IBSQoL Irritably bowel syndrome quality of life (score)

MRC Medical Research Council

MYMOP Measure yourself medical outcome profile

RCT Randomised controlled trial.

## Competing interests

TT is employed as a homeopathic practitioner by the UK NHS.

## Authors' contributions

This study was conceived by TT. TT and MW shared equally in the design process. The majority of data was collected by TT and analysis was shared equally by TT and MW. MW made substantive revisions to various drafts of this manuscript which was prepared for publication by TT. Both authors have read and approved the final manuscript.

## Pre-publication history

The pre-publication history for this paper can be accessed here:


